# Morin Flavonoid Adsorbed on Mesoporous Silica, a Novel Antioxidant Nanomaterial

**DOI:** 10.1371/journal.pone.0164507

**Published:** 2016-11-03

**Authors:** Francisco Arriagada, Olosmira Correa, Germán Günther, Santi Nonell, Francisco Mura, Claudio Olea-Azar, Javier Morales

**Affiliations:** 1 Departamento de Ciencias y Tecnología Farmacéuticas, Facultad de Ciencias Químicas y Farmacéuticas, Universidad de Chile, Sergio Livingstone, 1007, Independencia, Santiago, Chile; 2 Departamento de Química Orgánica y Fisicoquímica, Facultad de Ciencias Químicas y Farmacéuticas, Universidad de Chile, Sergio Livingstone, 1007, Independencia, Santiago, Chile; 3 Institut Químic de Sarriá (IQS), University Ramón Llull, Via Augusta, 390, 08017, Barcelona, Spain; 4 Departamento de Química Inorgánica y Analítica, Facultad de Ciencias Químicas y Farmacéuticas, Universidad de Chile, Sergio Livingstone, 1007, Independencia, Santiago, Chile; Brandeis University, UNITED STATES

## Abstract

Morin (2´,3, 4´,5,7-pentahydroxyflavone) is a flavonoid with several beneficial health effects. However, its poor water solubility and it sensitivity to several environmental factors avoid its use in applications like pharmaceutical and cosmetic. In this work, we synthetized morin-modified mesoporous silica nanoparticles (AMSNPs-MOR) as useful material to be used as potential nanoantioxidant. To achieve this, we characterized its adsorption kinetics, isotherm and the antioxidant capacity as hydroxyl radical (HO•) scavenger and singlet oxygen (^1^O_2_) quencher. The experimental data could be well fitted with Langmuir, Freundlich and Temkin isotherm models, besides the pseudo-second order kinetics model. The total quenching rate constant obtained for singlet oxygen deactivation by AMSNPs-MOR was one order of magnitude lower than the morin rate constant reported previously in neat solvents and lipid membranes. The AMSNPs-MOR have good antioxidant properties by itself and exhibit a synergic effect with morin on the antioxidant property against hydroxyl radical. This effect, in the range of concentrations studied, was increased when the amount of morin adsorbed increased.

## 1. Introduction

The emerging discipline of nanomaterials intends to apply physical principles common in materials science to challenges in areas such as drug delivery, structure and properties of powders and manufacturing and processing of particle systems for use in novel formulations [1–3]. Nanoparticles (NPs) have been used to incorporate (by occlusion [4], non-covalent adsorption [5] and covalent attachment [6]) different molecules (drugs [7], photosensitizers [8], biomolecules [9], antioxidants [10], and others [11]) and to release them in a controlled way. Nanoparticles have frequently been prepared using biomolecules (proteins [12], lipids [13, 14]), polymers [15, 16], metals [17, 18] and oxides such as silica [19].

Silica (SiO_2_) is an attractive material for chemistry, medicine and pharmaceutical sciences because it is optically transparent, chemically inert, mechanically stable, fairly biocompatible, and its synthesis is relatively easy [20, 21]. Additionally, surface functionalization of silica is well-established using a wide variety of coating procedures [22–24]. Thereby, mesoporous SiO_2_ nanoparticles (MSNPs) coated with amino groups (AMSNPs) have been used for adsorption, stabilization and separation of carboxylic and phenolic compounds by hydrogen bond interactions [25, 26].

**Fig 1:**
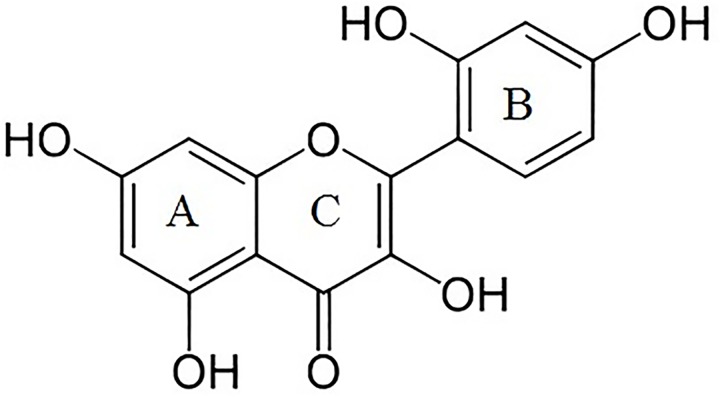
Chemical structure of morin.

Morin (2´,3,4´,5,7-pentahydroxyflavone) (Fig 1) is a phenolic compound present in vegetables and plants [27]. Several beneficial effects have been described, including anticancer [28], anti-inflammatory [29] and cardiovascular protective effects [30–32]. Moreover, morin (MOR) has shown interesting protective effects against UV-B radiation [33, 34], therefore its incorporation in topical formulation can be beneficial for skin health. However, its technological application (pharmaceutical and cosmetic formulations) is limited because this polyphenolic compound is sensitive to several environmental factors such as light, oxygen and pH among others [35].

Some studies have shown that morin is an efficient antioxidant owing to its ability to scavenge free radicals [36, 37] and quench singlet oxygen (^1^O_2_) [38–40]. The antioxidant capacity of polyphenols has been determined in different nanoparticles by evaluating the scavenging of 2,2-diphenyl-1-picrylhydrazyl (DPPH) free radical [41, 42]. Despite the potential benefits of using SNPs as vehicles for antioxidants, there are no studies describing effects of silica-bound morin or other flavonoids on ^1^O_2_ and hydroxyl radical (HO•) scavenging.

In this work, we synthetized morin-modified silica nanoparticles (AMSNPs-MOR) as useful antioxidant nanomaterial. Specifically, we characterized morin adsorption kinetics and isotherm onto AMSNPs and the antioxidant capacity of the resulting nanostructured material as HO• scavenger and ^1^O_2_ quencher.

## 2. Materials and Methods

### 2.1. Materials

Morin dihydrate was purchased from Merck. All chemicals (cetyltrimethylammonium bromide (CTAB) (Aldrich), ammonium hydroxide (NH_4_OH) (J.T. Baker), hydrochloric fuming acid 37% (Merck), tetraethylortho-silicate (TEOS), 3-aminopropyltriethoxysilane (APTES) (Aldrich), glycerin (Merck), polyoxyethylene sorbitan monooleate (polysorbate) 80 (Aldrich), sodium lauryl sulfate (Winkler) and chloride benzalkonium (reagent grade)) were used as received without any further purification.

Rose Bengal (RB) and 5,5-dimethylpyrroline N-oxide (DMPO) were purchased from Sigma-Aldrich and hydrogen peroxide 30% v/v was obtained from Merck.

All solvents used were reagent grade or HPLC quality. Water was purified and deionized using a Milli-Q system.

### 2.2. Methods

#### 2.2.1. Synthesis and surface modification of mesoporous silica nanoparticles

**Mesoporous silica nanoparticles (MSNPs)** were synthesized using a modified Stöber method [43] by slowly adding 7.2 mL tetraethyl orthosilicate (TEOS) to a mixture of 120 mL ethanol, 44 mL deionized water, 80 mg CTAB and 7.2 mL ammonia solution in a thermostated round bottom flask at 60°C under stirring.; then stirring was stopped, allowing the reaction to proceed for 2 h. The resulting dispersion was washed with several portions of ethanol followed by centrifugation and resuspension of the MSNPs and then 8 mL of HCl 37% were added and kept under stirring at 60°C for 12 h. The resulting suspension was washed again with several portions of ethanol followed by centrifugation and resuspension. The final MSNPs obtained were re-suspended in ethanol.

**Aminopropyl-modified silica nanoparticles (AMSNPs)** were prepared by treating MSNPs with (3-aminopropyl) triethoxysilane (APTES). MSNPs were suspended in 200 mL toluene and 0.8 mL of APTES were added at room temperature. The mixture was then heated to reflux for 24 h and allowed to cool. The nanoparticles were washed and centrifuged several times with portions of toluene, toluene: ethanol and ethanol and stored re-suspended in ethanol.

#### 2.2.2. Morin adsorption

Adsorption experiments were carried out in batch mode in triplicate to obtain the equilibrium and kinetic data [44, 45]. Preliminary experiments were done to figure out the optimal conditions for batch adsorption. A morin stock solution of 2 mg mL^-1^ was prepared and different desired volumes were taken and added to 30 mL of AMSNPs suspension in a flask. After addition of morin aliquot, the suspensions were stirred at 100 rpm until adsorption equilibrium was reached and subsequently centrifuged to 8000 rpm for 20 min to obtain suitable aliquots for analysis of residual morin concentration by an HPLC method previously developed and validated [46]. Thus, AMSNPs-MOR or just NP-MOR were obtained.

#### 2.2.3. Adsorption isotherms

Isotherm determination experiments were carried out by adding a fixed amount of morin solution (0.514 mg MOR /mL EtOH) to varying amounts of AMSNPs (15 mg—100 mg) in a volumetric flask (25 mL) to obtain a final morin concentration of 1x10^-4^ M; then the flask contents were stirred until adsorption equilibrium was reached. The amount of morin adsorbed at equilibrium onto the nanoparticles surface was calculated from the following equation [47]:

$$q_{e}=\frac{(C_{0}-C_{e})V}{m}$$ Eq 1

Where *q*_e_ is the amount of morin adsorbed per unit amount of AMSNPs nanoparticle at equilibrium (given in mg g^-1^), *C*_0_ and *C*_e_ are the morin concentrations in solution before and after adsorption, respectively (mg L^-1^), *V* is the bulk volume of the medium (L) and *m* is the mass of nanoparticles (g).

#### 2.2.4. Adsorption kinetics

The kinetics of morin adsorption onto AMSNPs was assessed by adding an aliquot of morin solution (2 mg mL^-1^) to nanoparticle suspensions (1.2 g mL^-1^). The suspension was kept at 25°C under stirring. At several time intervals, samples were collected, centrifuged, and the concentration of morin in the supernatant was determined by HPLC. The amount of morin adsorbed per unit amount of nanoparticle after each time interval *q*_*t*_ (mg g^-1^), was calculated by the following equation:

$$q_{t}=\frac{(C_{0}-C_{t})V}{m}$$ Eq 2

Where C_0_ is the initial concentration of morin (mg L^-1^) and C_t_ is the concentration at time “t” (mg L^-1^), *V* and *m* having the same meaning as above.

#### 2.2.5. Nanoparticle characterization

The average particle size, polydispersity index and Zeta potential of MSNPs, AMSNPs and AMSNPs-MOR were analyzed using a Malvern Zetasizer Nano ZS90 (Malvern, UK). The samples were diluted with ethanol and different pH buffers. Morphological characterization was based on scanning electron microscopy (SEM) FEI^TM^, inspect F50 model, where the samples were placed on a silicon wafer grid and then coated with gold. Fourier transform infrared (FT-IR) spectra were obtained on an Interspec 200-X FT-IR spectrometer with 4 cm^-1^ resolution in the wavenumber range of 4000–400 cm^-1^ and 16 scans were taken with the average from each spectrum.

#### 2.2.6. Morin removal from nanoparticles

In order to evaluate the removal of morin from the nanoparticles-morin caused by different components frequently used in commercial pharmaceutical and cosmetic formulations, such as glycerin (5% and 10%), polysorbate 80 (0.5% to 5%), sodium lauryl sulfate (0.05% to 0.5%), and benzalkonium chloride (0.01% to 0.1%), AMSNPs with adsorbed morin (AMSNPs-MOR or just NP-MOR) were suspended in 10 mL of each of the above mentioned solutions and kept in contact under gentle stirring until equilibrium was reached; then, the suspension was centrifuged and the concentration of morin in solution was quantified by HPLC [46].

#### 2.2.7. Singlet oxygen quenching

The total quenching rate constant (*k*_T_) for the deactivation of ^1^O_2_ by AMSNPs-MOR was determined in D_2_O suspensions by monitoring the time-resolved phosphorescence of ^1^O_2_ following laser excitation of Rose Bengal (RB) electrostatically adsorbed onto AMSNPs (NP-RB).

Time-resolved ^1^O_2_ near-infrared phosphorescence was measured by means of a PicoQuant FluoTime 300 fluorescence lifetime spectrometer. A PLS 575 LED-head was employed as the pulsed light source, in burst mode. Luminescence of singlet oxygen was monitored at 1270 nm using a Hamamatsu NIR-PMT detector (H10330-45) and analyzed with PicoQuant’s Fluofit software.

#### 2.2.8. Hydroxyl radical scavenging

The antioxidant capacity of AMSNPs-MOR against the hydroxyl radical (HO•) was assessed by spin-trapping using DMPO [48]. HO• was generated by photolysis of hydrogen peroxide using a UV-Vis-NIR light source model DH-2000-BAL purchased from Ocean Optics. Solutions of DMPO (200 mM) and of H_2_O_2_ (10% v/v) were prepared in Milli Q water. Morin was dissolved in a solution of glycerin 10% v/v with a small quantity of ethanol as co-solvent (0.2% of the final volume) to a final concentration of 0.11 mM. The control solution was prepared with DMPO 200 mM (50 μL), hydrogen peroxide 10% (50 μL) and glycerin 10% (50 μL). Due to the low adsorption efficiency (11 mg g^-1^), the experimental concentration range achievable is limited and two concentrations of morin were selected (18 and 36 μM). The samples were prepared replacing the glycerin volume by a morin solution 0.11 mM (solution 1), AMSNPs solution 6800 mg L^-1^ (solution 2) and AMSNPs-MOR solution 0.11 mM (solution 3). In order to evaluate the presence of photolytic degradation paths (in absence of hydroxyl radical), an assay with morin was performed, observing the formation of a DMPO-HO• adduct in a small quantity (10% of the signals in the control spectrum), which was discounted on the intensity of the signals in all the measurements.

EPR spectra were recorded at the X band (9.81GHz) using a Bruker ECS106 spectrometer with a rectangular cavity and 50 kHz field modulation. Spectrometer conditions were: microwave frequency 9.81 GHz, microwave power 20 mW, modulation amplitude 0.91 G, receiver gain 59 dB, time constant 81.92 ms and conversion time 40.96 ms [49]. Each spectrum was obtained after 10 scans.

The antioxidant capacity was considered as directly proportional to the decrease in the area of the signal in comparison to a control spectrum, which did not contain any antioxidant.

$$Antioxidant\;capacity=\frac{A_{CONTROL}-A_{SAMPLE}}{A_{CONTROL}}\times 100$$ Eq 3

Where *A*_CONTROL_ is the area under a peak in the control spectrum, *A*_SAMPLE_ is the area under a peak in the spectra which an antioxidant (morin, AMSNPs or AMSNPs-MOR) is present.

### 2.3. Statistical analysis

Data are presented as mean ± SD of *n* independent experiments. Statistical analysis was performed using a R^2^ parameter, Chi-Square test or one-way ANOVA and comparisons between groups were performed by Tukey`s multiple comparison test. p < 0.05 was considered significant.

## 3. Results and Discussion

### 3.1. Characterization of NPs

The changes in size were observed in the synthesis and upon addition of morin to 100 mg of AMSNPs, showing that morin was incorporated onto silica nanoparticles. The mean particle size of MSNPs, AMSNPs and AMSNPs-MOR obtained by Dynamic Light Scattering determinations were 105, 125 and 155 nm, respectively (values consistent with SEM images). Polydispersity indices ranged from 0.02 to 0.17 revealing high size homogeneity of these NPs. Previously, the mean pore size of MSNSPs and AMSNPs determined by TEM was about 3 nm (data not shown), similar pore size has been reported in other works [50–52]

**Fig 2:**
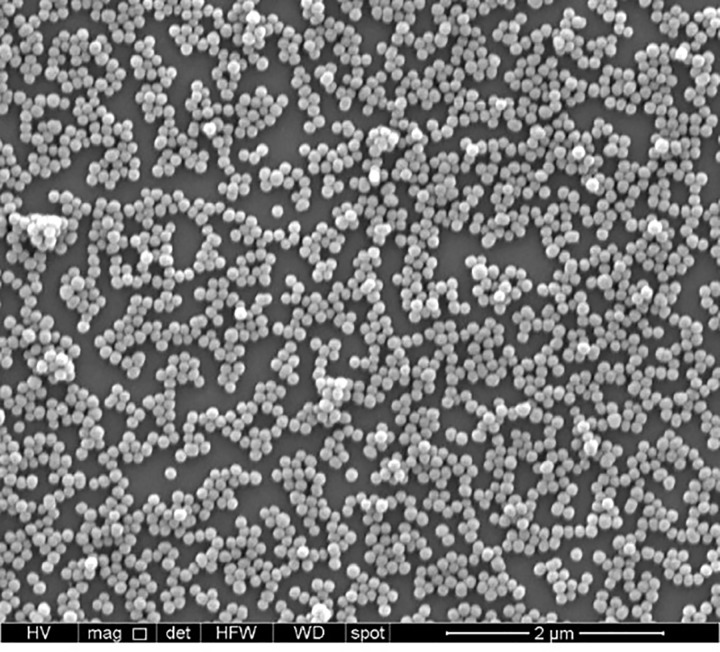
SEM micrograph of AMSNPs-MOR.

A SEM image in Fig 2 shows the morphology of the AMSNPs-MOR. All NPs observed in SEM images were of similar size, showing a monodisperse sample.

Table 1 shows the results of zeta potential measurements for AMSNPs and AMSNPs-MOR (2 mg MOR / 100 mg NP) dispersed in solutions at pH 1, 3, 7 and 9. At pH 1 and 3 morin adsorbed on the nanoparticles does not change their positive Zeta potential value, possibly due to molar ratio between OH of morin structure and NH_2_ of NP is not equivalent, therefore the effect of this flavonoid on the surface potential is not relevant. Moreover, at pH 7 and pH 9, morin is ionized (phenolate form), changing the nanoparticle surface potential (negative). The ionization of hydroxyl groups of flavonoids is highly dependent on their position, p*K*_a_ and the medium pH. At pH 7, only the hydroxyl located at position 2´ of the morin’s B ring is ionized [53].

**Table 1:** Zeta potential values of NPs dispersed in aqueous solutions at different pH.

Nanoparticles	Zeta potential (mVolts)			
	pH 1	pH 3	pH 7	pH 9
AMSNPs	+41.8	+27.6	-1.5	-5.2
AMSNPs-MOR	+44.7	+26.2	-9.5	-17.7

**Fig 3:**
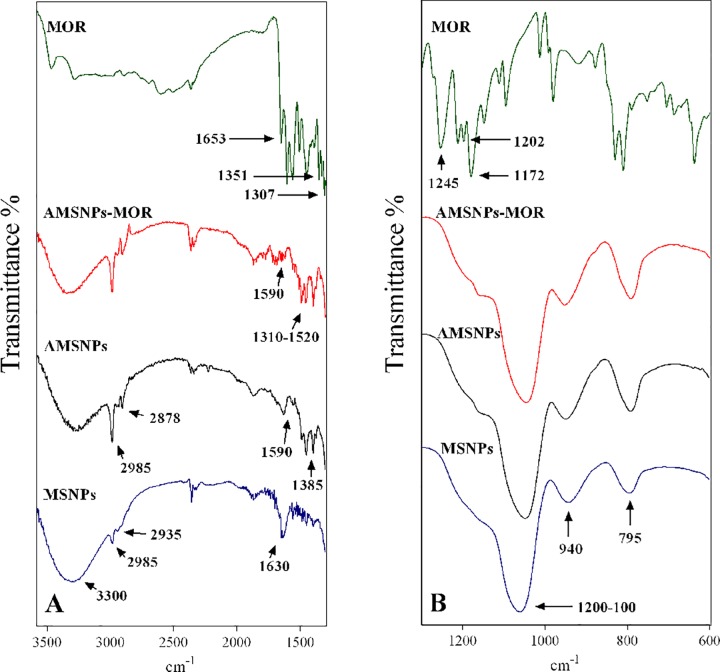
FTIR spectra. (A) MSNPs (blue), AMSNPs (black), AMSNPs-MOR (red) and morin (green) in the range of 3600–1300 cm^-1^ and (B) MSNPs (blue), AMSNPs (black), AMSNPs-MOR (red) and morin (green) in the range of 1299–600 cm^-1^.

FTIR spectra were recorded in the range of 4000–400 cm^-1^. The spectrum of morin (Fig 3A and 3B) reveals a band at 1653 cm^-1^ due to (C = O) stretching vibration, the (C-OH) deformation vibrations are observed at 1351 cm^-1^ and 1307 cm^-1^. The peaks at 1202 cm^-1^ and 1172 cm^-1^ corresponding to the (C-OH) stretching and the band located at 1245 cm^-1^ are attributed to the (C-O-C) bending. The MSNPs spectrum in the Fig 3B showed a broad band (1200–1000 cm^-1^) corresponding to the asymmetric vibration of group (Si-O-Si). Additionally, the bands at 940 cm^-1^ and 795 cm^-1^ are related to asymmetric vibration of Si-OH and symmetric vibration of Si-O, respectively. Fig 3A showed a very characteristic absorption band at 3300 cm^-1^ assigned to O-H stretching in H-bonded water, also supported by the presence of 1630 cm^-1^ band due to scissor bending of molecular water. Moreover, the presence of the bands at 2985 cm^-1^ (CH_3_) and 2935 cm^-1^ (CH_2_) is attributed to the presence of unreacted TEOS [54].

Fig 3 displayed the AMSNPs spectrum, which shows some changes when compared with MSNPs spectrum; the band corresponding to O-H stretching (3300 cm^-1^) of molecular water and the band of asymmetric vibration of Si-OH (940 cm^-1^) decreased their intensity, besides bands at 2985 cm^-1^ and 2878 cm^-1^ slightly increased due to the vibration of (CH_2_) group of the propyl chain and a new band at 1385 cm^-1^, corresponding to the Si-CH_2_ bending mode was observed, suggesting the presence of the amino group of APTES molecule in the terminal position of the propyl chain; also a new band assigned to NH_2_ asymmetric bending was observed at 1590 cm^-1^. This implies that the concentration of Si-OH groups in the surface has decreased and the amount of NH_2_ groups has increased, suggesting a successful APTES functionalization [55–58]. The IR spectrum of NP-MOR showed at least three significant differences in comparison with MSNPs and AMSNP spectra. First, in the region of 1310–1520 cm^-1^ some changes were observed due to superposition and/or shift of the bands corresponding to the C-C skeletal vibration of morin aromatic ring. Second, the band ascribed to the NH_2_ asymmetric bending diminished considerably, these findings suggest that morin was successfully incorporated onto silica nanoparticles through the interaction with NH_2_ group of APTES. Finally, the bands centered at 3300 cm^-1^ increased their intensity, because of the morin–OH groups, present now in nanoparticles surface, observation in agreement with other authors. These results suggest that interaction between nanoparticles and morin, involves NH_2_ group of APTES and OH group of A or C ring of morin. In fact, Vergara-Castañeda et al. [59] reported the linking of quercetin onto silica nanoparticles, they state that quercetin conjugation in the NPs involves a covalent-like bond through amide groups of APTES-NPs with OH and/or C = O groups of quercetin. Nevertheless, more tests are necessary to unravel the exact way by which incorporation takes place. On the other hand, different antioxidant measurements allow us to elucidate the possible point of linkage, considering the different antioxidant capacity of morin functionalities.

### 3.2. Adsorption properties

**Fig 4:**
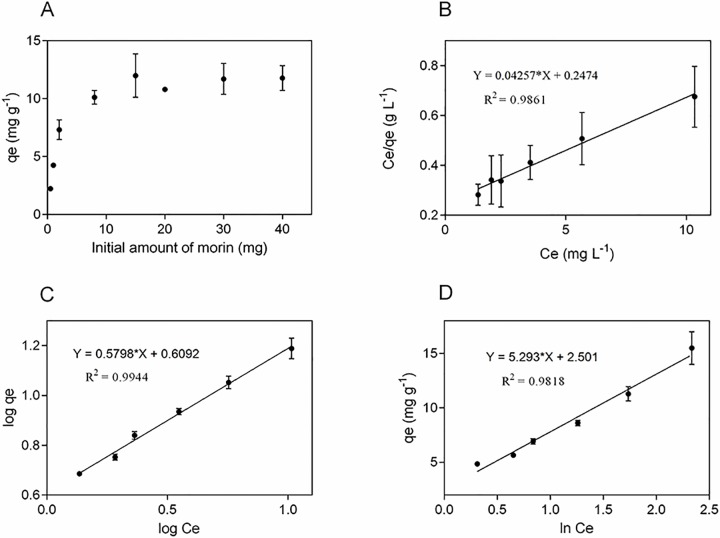
Morin adsorption onto silica nanoparticles. (A) Adsorbed amount of morin as a function of different initial amount of morin; linear fit of experimental data obtained using (B) Langmuir (C) Freundlich and (D) Temkin isotherm models at 25°C. Values are the mean of n = 3 (mean± SD).

It is well known that the adsorption capacity of a surface is frequently determined by the degree of compatibility between adsorbent and adsorbate [60]. This is important because the interaction between adsorbate and adsorbent is fundamental for the design of the adsorption system [61]. For batch adsorption experiments, the morin concentration was 5.9 x 10^−3^ M. Fig 4A shows the data obtained (in triplicate) of the adsorbed amount of morin onto nanoparticles versus the initial amount of morin. The maximum adsorption obtained was 11 mg g^-1^. The adsorbed amount increases when the initial amount of morin increases, reaching saturation at initial morin values above 8–10 mg.

Higher adsorption values were obtained compared with other reports about flavonoid adsorption onto silica nanoparticles [42]. This must be due to surface functionalization with APTES, as reported by Berlier et al. [62] using rutin; these authors hypothesized that it is possible that the aminopropyl chains play an important role in the van der Waals interaction with the quercetin aglycone. It is clear that the type of functionalization plays a pivotal role in the adsorption capacity; it also is affected by the physicochemical and structural characteristics of the adsorbed molecule. Previous experiments in our laboratory showed a decrease in the adsorption of quercetin on silica nanoparticles upon functionalization with APTES, around half of that obtained with morin (data not shown).

### 3.3. Adsorption isotherms

The function that expresses the magnitude of the retention and behavior of a molecule on a solid surface once the equilibrium of adsorption/desorption phenomena have been reached, it can be described from the relationship between the remaining concentration of a molecule with the concentration or amount of the same compound retention on the surface at constant temperature, this relationship is commonly known like as Adsorption Isotherm [44, 63].

The Langmuir [64, 65], Freundlich [66] and Temkin [67] isotherm models are often used to describe the adsorption equilibrium and provide an approach to elucidate the adsorption mechanism, the surface properties and also the degree of affinity of the adsorbent for the adsorbate. Langmuir model assumes a monolayer adsorption, this suggests that the adsorption occurs at a finite number of localized sites, with no lateral interaction and steric hindrance between the adsorbed molecules; according to this, exists a maximum adsorption when a saturated monolayer of molecules is produced on the surface. On the other hand, Freundlich is an empiric model that describes the non-ideal and reversible multilayer adsorption, applicable to heterogeneous surfaces with non-uniform distribution of adsorption heat and intensity of affinity over the surface. Temkin model considers the effects of some indirect adsorbate-adsorbate interactions on adsorption isotherms and suggests these interactions would decrease linearly the heat of adsorption with the increase of coverage.

In order to determine the maximum adsorption capacity of the nanoparticles and to better describe the relationship between adsorbent and adsorbate at equilibrium conditions, we analyzed our data according to the linearized forms of Langmuir, Freundlich and Temkin adsorption isotherms, which correspond to the following equations:

$$\frac{C}{q_{e}}=\frac{1}{Q_{m}b}+\frac{1}{Q_{m}}c$$ Eq 4

$$\log(q_{e})=\log\;K_{f}+\frac{1}{n}\log\;C$$ Eq 5

$$q_{e}=B\;\ln\;A+B\;\ln\;C_{e}$$ Eq 6

Where *q*_e_ is the equilibrium concentration of morin on the solid surface phase (mg g^-1^), *C* is the concentration of morin in solution (mg L^-1^), *Q*_m_ is the maximum monolayer uptake by the nanoparticles (mg g^-1^), and *b* is the Langmuir constant for the equilibrium adsorbate-adsorbent (L mg^-1^). *K*_f_ is the Freundlich coefficient ((mg g^-1^)(L mg^-1^)^1/*n*^), where *n* is the Freundlich constant (index of adsorption intensity or surface heterogeneity) which denotes a favored adsorption if the value lies between 1 and 10 (chemisorption process is favored and the surface is more heterogeneous when slope, 1/*n*, value approaches zero) [66]. *A* is the Temkin isotherm constant (L·mg^-1^), *B* is a constant defined as $B=\frac{RT}{b_{T}}$, where *T* is the absolute temperature in Kelvin, *R* is the universal gas constant (8.3143 J mol^-1^ K^-1^) and *b*_*T*_ is a constant related to the heat of adsorption.

The experimental data could be well fitted with Langmuir, Freundlich and Temkin isotherm models (Fig 4), showing a high coefficient of determination for three models (R^2^_Freundlich_ = 0.9944; R^2^_Langmuir_ = 0.9861; R^2^_Temkin_ = 0.9818).

While the three models seem to appropriately describe the data, the high correlation of the data with the Freundlich model suggests that it is most suitable to fit. The above results suggest that morin adsorption onto the surface of the nanoparticles is not restricted to the formation of a monolayer but follows a multilayer adsorption, with non-uniform distribution of adsorption heat and affinities over the heterogeneous surface [44, 67]. On the other hand, the coefficients of determination coefficients for the Langmuir and Temkin isotherm models are statistically significant, indicating that the adsorption process also involves the formation of a monolayer of morin. This could be explained because the adsorption-desorption equilibrium can be a complex process controlled by more than one mechanism, where dimer formation and possible self-assembly of morin in piled up form [68], could generate multilayers at the surface, involving electrostatic forces. The parameter *n* equal to 1.72, higher than 1, indicates a favorable adsorption process.

Based on the non-linear Chi-square statistical analysis [69], we found that the data fit better to the Langmuir model although the R^2^ is smaller in comparison to the Freundlich model, these results suggest a better concordance with the experimental data, in fact, the maximum monolayer uptake value (23.5 mg g^-1^) was calculated from the equation of the linearized Langmuir isotherm, this value was consistent with the experimental value of the maximum adsorption, determined from the plateau in Fig 4.

Furthermore, the feasibility of the adsorption can be elucidated from the dimensionless constant, commonly known as separation factor (R_L_) [70], and can be represented as:

$$R_{L}=\frac{1}{1+bC_{0}}$$ Eq 7

Where *b* is the Langmuir constant (L mg^-1^) and C_0_ is the initial concentration of morin (mg L^-1^). R_L_ values indicate the nature of the adsorption for an unfavorable reaction (R_L_> 1), linear case (R_L_ = 1), favorable process (0 < R_L_< 1) and irreversible case (R_L_ = 0). The R_L_ values were 0.26–0.009 at 25°C, suggesting that the adsorption process is favorable. The values of the different parameters obtained from the linearized isotherm models are summarized in Table 2.

**Table 2:** Adsorption isotherms parameters of morin onto AMSNPs.

Isotherm	Equation	Parameters
Langmuir	(C/q_e_) = (1 /(Q_m_ b)) + (1/ Q_m_) C	Q_m_ (mg g^-1^) = 23.5
		b (L mg^-1^) = 0.172
		R^2^ = 0.9861
		R_L_ = 0.25–0.009
		X^2^ = 34.5
Freundlich	log(q_e_) = log K_f_ + (1/n) log C	K_f_ ((mg/g)(L/mg)^1/n^) = 4.1
		n = 1.72
		R^2^ = 0.9944
		X^2^ = 564.1
Temkin	q_e_ = B ln A + B ln C_e_	B = 5.293
		A (L mg^-1^) = 1.6
		R^2^ = 0.9818
		X^2^ = 69.1

### 3.4. Adsorption kinetics

The study of adsorption kinetics is crucial because it can unravel both the adsorption and the desorption mechanisms [61]. The rate of adsorption may be determined by mechanisms such as chemical reaction, mass transfer, diffusion control or a combination of them [71]. Since adsorption is a time-dependent process, it is useful to determine its rate in order to evaluate or design the adsorbent [72].

**Fig 5:**
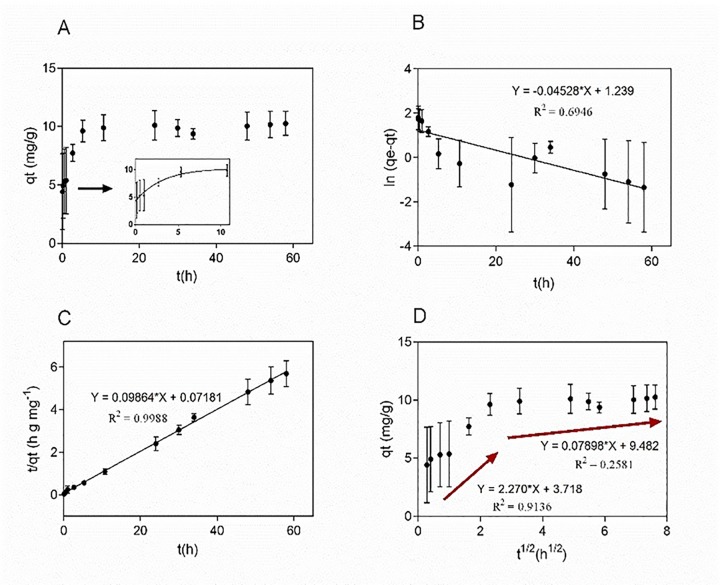
Adsorption kinetics of morin onto AMSNPs. (A) Effect of contact time; (B) linear fit of experimental data obtained using pseudo-first order model, (C) pseudo-second order model and (D) intra-particle diffusion model. Values are the mean of n = 3 (mean± SD).

Fig 5A shows the effect of contact time, maximum adsorption is reached after 10 h during a gradual adsorption process. After that, a second stage was observed leading to a final equilibrium state, possibly due to a decrease in the number of available adsorption sites, as well as to the decrease in the concentration of morin in solution phase.

Various kinetics models have been suggested to describe the adsorption process, and new derivation methods have been proposed to optimize established models to provide a better understanding of the various processes and to establish conditions that limit the use of each model; although results are debatable, valuable approaches are presented for the various theoretical viewpoints of the models [61, 73, 74]. Considering this, the kinetics of morin adsorption onto the nanoparticle surface was analyzed using three different kinetic models: the pseudo-first-order proposed first by Lagergren (Eq 8) [75], the pseudo-second-order models of Ho and McKay [76] (Eq 9) and Weber and Morris intra-particle diffusion model (Eq 10) [77]:

$$\ln(q_{e}-q_{t})=\ln\;q_{e}-k_{1}t$$ Eq 8

$$\frac{t}{q_{t}}=\frac{1}{k_{2}{q_{e}}^{2}}+\frac{t}{q_{e}}$$ Eq 9

qt=Kidt12+I Eq 10

Where *q*_e_ and *q*_t_ are the amount of adsorbed morin onto nanoparticle surface at equilibrium and at time *t* (mg g^-1^), respectively; *k*_1_ is the observed rate constant of the pseudo-first order model (h^-1^) and *k*_2_ is the observed rate constants of the pseudo-second-order model (g mg^-1^ h^-1^). *K*_id_ is the intra-particle diffusion rate constant (g/mg h^1/2^) and *I* is related with the thickness of the boundary layer.

Fig 5B and 5C show the linear fit to pseudo-first order and pseudo-second-order equation, respectively. *k*_1_ was obtained from the slope of plot ln(*q*_e_−*q*_t_) versus *t; k*_2_ was obtained from the intercept and slope of plot *t/q*_t_ versus *t*. The *q*_*e*_ value obtained with pseudo-first-order model is far from the experimentally found value, suggesting that the model, despite the good fit, does not describe the process, on the other hand, *q*_e_ value given by the pseudo-second-order treatment is much closer to that found experimentally. The values of *R*^2^ suggest that the pseudo-second-order model is more suitable to predict the kinetic mechanism for the adsorption of morin onto silica nanoparticles in the present work.

The stages of adsorption process are studied thoroughly with Weber and Morris intraparticle diffusion model which is observed in Fig 5D. According to the Eq (10), if the plot gives a straight line, then the adsorption is controlled solely by the intraparticle diffusion, but the adsorption data present a multi-linear plot, a gradual adsorption following by the final equilibrium of the process, showing that there are more than one step involved in the adsorption process [78]. All results suggest a complex process where rate-limiting step of adsorption could be a chemisorption rather than an intra-particle diffusion only. Only a few studies involving adsorption of flavonoids onto silica nanoparticles for pharmaceutical and/or cosmetic purposes have been reported, and even less works depict the kinetic process of morin on such a pharmaceutical carrier. Other works about kinetic processes involving silica nanoparticles obtained similar results to those reported here, namely that the pseudo-second order model is more suitable to predict the behavior of different compounds adsorbed onto AMSNPs in that case [79–81]. The data obtained from Eqs 8 to 10 are summarized in Table 3.

**Table 3:** Adsorption kinetics parameters of morin onto AMSNPs.

Kinetics model	Equation	R^2^	Parameters
Pseudo first-order	ln (q_e_—q_t_) = - 0.04528 t + 1.239	0.6946	k_1_ = 0.04528 h^-1^
			q_e_ = 3.45 mg g^-1^
Pseudo second-order	(t / q_t_) = 0.09864 t + 0.07181	0.9988	k_2_ = 0.136 g mg^-1^h^-1^ q_e_ = 10.14 mg g^-1^
Intra-particle diffusion	q_t_ = 2.27 t^1/2^ + 3.718	0.9136	k_id1_ = 2.27 mg g^-1^h^-1/2^ I_1_ = 3.718 mg g^-1^
	q_t_ = 0.07898 t^1/2^ + 9.482	0.2581	k_id2_ = 0.07898mg g^-1^h^-1/2^ I_2_ = 9.482 mg g^-1^

### 3.5. Morin removal from nanoparticles

**Fig 6:**
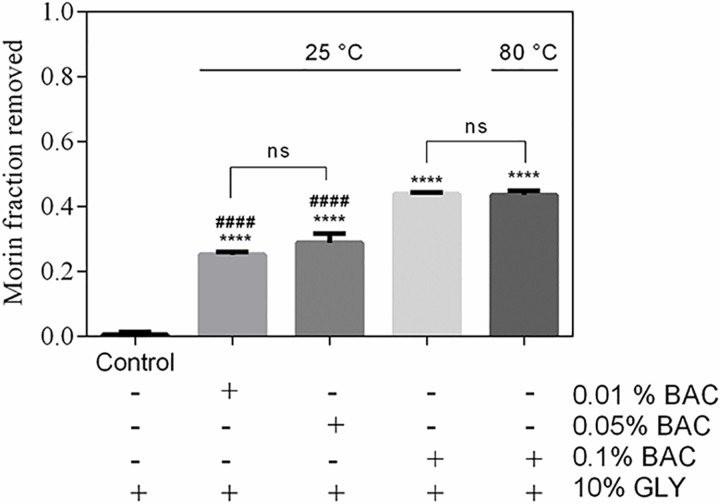
Removal of adsorbed morin by benzalkonium chloride (BAC). Values are the mean of n = 3 (mean± SD). ****p < 0.0001 vs. control, ^####^p < 0.0001 vs. 0.1% BAC at 25°C or 0.01% BAC at 80°C.

The morin removal from AMSNPs-MOR by effect of the presence of a polyol compound (glycerin) and anionic (sodium lauryl sulfate), cationic (benzalkonium chloride) and nonionic (polysorbate 80) surfactants was determined. Glycerin (GLY) solutions at 5% and 10% did not generate an effect on morin desorption (or removal). Polysorbate 80 solutions at 0.5%, 1%, 2% and 5% had a negligible effect on the removal. The removal generated by the solutions of sodium lauryl sulfate (SLS), did not exceed 5% at each concentration. Moreover, removal of morin by the benzalkonium chloride (BAC) was more significant, reaching average values of 44% of morin desorption in different tested concentrations, with a tendency to increase the desorption as the concentration increases (Fig 6).

These results can be explained because of the slight negative charge on the adsorbed morin which would interact with benzalkonium chloride (positively charged), causing increased desorption respect to the other components under study. It is interesting to mention that when the AMSNPs-MOR system is in contact with the different media studied, morin removal occurs only once, i.e. morin that is not removed is irreversibly adsorbed when the dispersion medium are those mentioned above. In order to force desorption of morin, we performed desorption experiments at 80°C; nevertheless, removal of morin was negligible and morin layer onto silica nanoparticles remained irreversibly adsorbed. This is consistent with the results discussed from isotherms and kinetic models. To discard the idea that the adsorption occurs in a priority way in the pore; we previously performed experiments with non-porous silica nanoparticles. Nevertheless, no significant differences in the adsorption compared with our porous silica nanoparticles were found. Thus, we hypothesized that removal of morin take place mainly on the nanoparticles surface.

Taken together, all results suggest a first layer of morin chemisorbed onto the nanoparticles; while external layers (association or self-assembly of morin in piled up form) are physisorbed and easily removed by typical components of pharmaceutical formulation e.g. benzalkonium chloride. Similar results has been mentioned by others authors, finding that the most probable route for flavonoid incorporation in the AMSNPs is by a conjugation based in covalent-like bonding [59].

### 3.6. Antioxidant capacity studies

#### 3.6.1. Deactivation of singlet oxygen by AMSNPs-MOR

The singlet oxygen (^1^O_2_), an electronically excited species of oxygen, can be generated in biological systems usually by two different routes; by reactions known as “light reactions” and “dark reactions” produced by process like photo-excitation and chemi-excitation respectively [82]. In states of oxidative stress in the skin, various ROS are generated, including ^1^O_2_, which can significantly alter different biomolecules like proteins, lipids and DNA, with undesirable consequences for health [83]. Therefore, deactivation of singlet oxygen, could help to avoid or reduce such kind of adverse effects.

**Fig 7:**
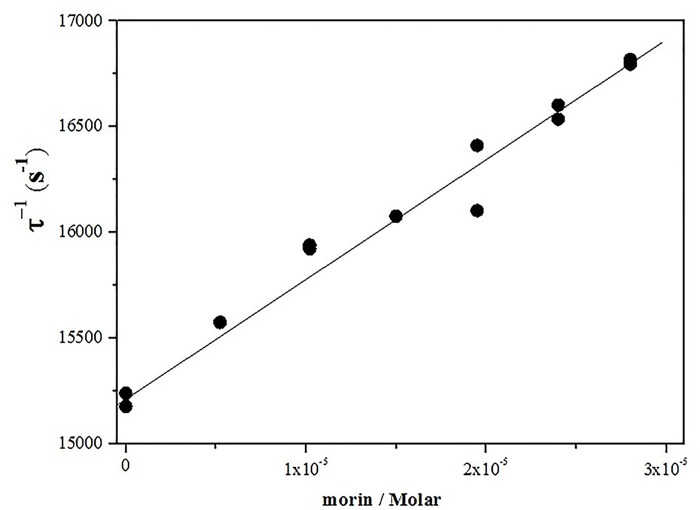
Stern–Volmer type plot for singlet oxygen deactivation by AMSNPs-MOR.

The deactivation of singlet oxygen with a flavonoid or other quencher, involves physical quenching (deactivation) and/or chemical (reactivity) processes. The sum of the physical quenching (*k*_q_) and chemical reaction (*k*_R_) constants correspond to the total rate constant (*k*_T_) [38, 39]. In this work, *k*_T_ values for the deactivation of singlet oxygen by AMSNPs-MOR dispersed in D_2_O were obtained by measuring the first-order rate of singlet oxygen of luminescence decay in the presence and absence of morin (Eq 11).

$$\tau^{-1}=\tau_{0}^{-1}+k_{T}[MOR]$$ Eq 11

where τ^-1^ is singlet oxygen lifetime in presence of morin and τ_0_^-1^is singlet oxygen lifetime in its absence (τ_0_^−1^ = 1/k_d_). Values of *k*_T_ were calculated from slopes of τ^−1^ vs. [MOR] plots. Fig 7 shows the corresponding Stern-Volmer plot.

The obtained value of *k*_T_ (4.5 x 10^7^ M^-1^s^-1^) indicates that morin is still an efficient quencher of singlet oxygen when it is immobilized on the silica surface. However this value is one order of magnitude lower than that in homogeneous media (1.3 x 10^8^ M^-1^s^-1^ in D_2_O pD 7.4) and erythrocyte ghost membranes dispersed in D_2_O pD 7.4 (1.4x10^8^ M^-1^s^-1^)[38]. This could be explained by the interaction between the most acidic hydroxyl moiety of morin (the 2´-OH group on the B ring, pKa 5.2), with the amine group of the NP [53], the formation of dimers and other aggregates of morin on the NP surface, and the slower oxygen diffusion on the solid nanoparticle compared to neat solvents and lipid membranes. In solid organic polymers, the bimolecular quenching rate constants (*k*_q_) for deactivation of singlet oxygen by efficient quenchers are smaller than in homogeneous solutions because quenching process in polymers is mainly controlled by solute diffusion to yield the singlet oxygen-quencher encounter pair [84]. Indeed this reduction in reactivity would be compensated with the higher availability of quencher species, resulting in a potential promissory system for real applications

#### 3.6.2. Reactivity of AMSNPs-MOR against the hydroxyl radical

Consequence of its function as a barrier, the skin is a potential target for oxidative stress due to production of free radicals from different sources. These free radicals threaten the integrity of skin as they do to other tissues [85, 86], but the skin is at risk because its exposition to oxygen from the inside at levels provided by the blood and from the outside at higher levels provided by the air, and the continuous exposition to light [34], which generates hydroxyl radicals through breakage of hydrogen peroxide (H_2_O_2_ + UV radiation → 2 HO•). Free radicals can also be formed by the presence of xenobiotics that can contain ions Fe(II) or Cu(I) that act as a catalyst of hydrogen peroxide decomposition (H_2_O_2_ + Fe(II)/Cu(I) → HO• + HO^-^ + Fe(III)/Cu(II)).

Morin has shown antioxidant activity against the damage caused by hydroxyl radical, through a proton-coupled electron transfer (PCET mechanism) [87, 88], where the more reactive functional groups in the molecule are the resorcinol moiety in B-ring, or by forming a chelation site for iron ions between 2´-hydroxyl group of ring B and 3-hydroxyl group of ring C [89].

**Fig 8:**
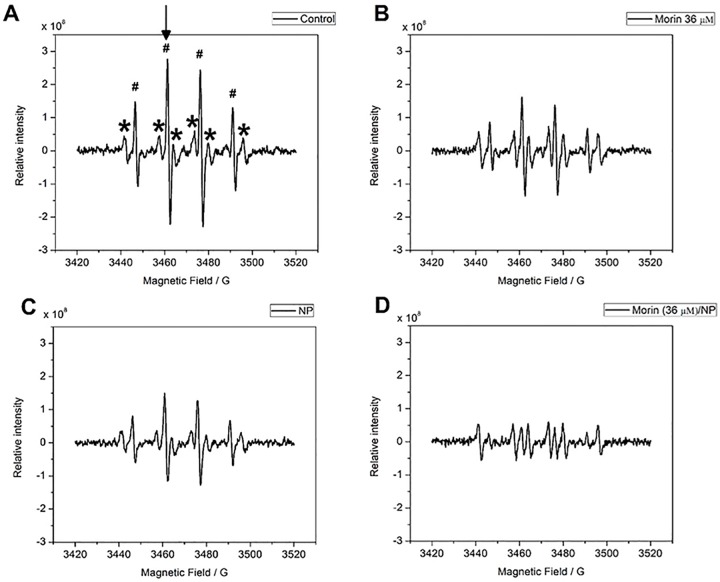
EPR spectra. Control (DMPO + H_2_O_2_+glycerin 10%); (B) morin (DMPO + H_2_O_2_ + morin 0.11 mM/glycerin 10%); (C) AMSNPs (DMPO + H_2_O_2_ + NP 6800 mg L^-1^/glycerin 10%); (D) AMSNPs-MOR (DMPO + H_2_O_2_ + morin 0.11 mM/NP 6800 mg L^-1^ /glycerin 10%). In spectrum (A), the symbol **(#)** indicates the presence of DMPO-HO• adduct. The symbol (*) indicates the presence of the DMPO/glycerin adduct; and the arrow indicates the peak that was used for the analysis of each spectrum.

The antioxidant reactivity of AMSNPs-MOR against hydroxyl radical was studied in a system where this radical was generated in situ by hydrogen peroxide 10%v/v UV-photolysis [90, 91]. As described before, morin has been identified as a potent antioxidant against hydroxyl radical [92], but there is no reported information about its antioxidant capacity when absorbed onto silica NPs. The presence of glycerin in the medium generated a hyperfine splitting pattern of six signals that performed a constant intensity in all the spectra, for this reason the analysis became independent of the presence of glycerin (Fig 8A).

**Fig 9:**
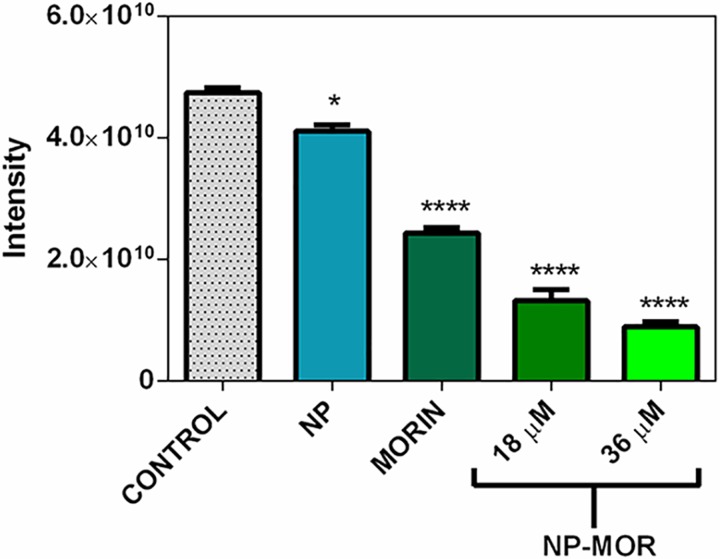
Antioxidant capacity against HO• radical. Control (0 μM morin), AMSNPs (0 μM morin), morin solution suspended in 10% glycerin (MORIN) (36 μM morin), AMSNPs-MOR (18 μM and 36 μM). All the values are given as average ± SD, in three independent assays (*p < 0.05 vs. control; ****p < 0.0001 vs. control).

To evaluate the scavenging activity of the AMSNPs-MOR, the formation of the DMPO- HO• adduct by every component was measured; the results show that the NP possess scavenging capacity against hydroxyl radical (Fig 8C). On the other hand, the scavenging capacity of AMSNPs-MOR (Fig 8D) increases in 57% in comparison to morin (at the same concentration) (Fig 8B), which indicates the existence of a synergic effect between the NP and the adsorbed-morin. This synergistic effect has also been observed between SiO_2_ nanoparticles and covalently-linked-gallic acid derivative against DPPH• radical [41]. As the antioxidant activity of morin did not decrease when is adsorbed onto the nanoparticles, this may indicate that the resorcinol moiety in B-ring, which is the most reactive site in the molecule against free radicals, is able to interact with hydroxyl radicals. For quercetin, which is structurally similar to morin by replacing the resorcinol in B-ring to a 3’,4’-cathecol moiety, when adsorbed onto silica NP, it has been documented a similar behavior against superoxide radical [93], indicating that the B-ring is free to react with the free radicals and that the linkage of this flavonoids to the NP takes place through the polar groups in A and C-rings [59, 94]. The antioxidant capacity against hydroxyl radical is summarized in Fig 9.

## 4. Conclusions

The aim of this study was to evaluate the antioxidant capacity and the kinetics and the adsorption isotherm of morin onto silica nanoparticles. Mesoporous and spherical aminopropyl-modified silica nanoparticles (~150 nm) were prepared and characterized. The maximum adsorption capacity of these nanoparticles for morin (20 mg g^-1^) was performed by Langmuir, Freundlich and Temkin adsorption isotherms.

The kinetics of morin adsorption onto nanoparticle surface was analyzed using three different kinetic models, the pseudo-first-order proposed first, pseudo-second-order models and intra-particle diffusion model. The results suggest that the process is clearly complex and is more likely that the rate-limiting step of this adsorption system can be a chemisorption rather than an intra-particle diffusion only.

The antioxidant properties of AMSNPs-MOR by monitoring of singlet oxygen deactivation and OH radical scavenging were studied. The total quenching rate constant (k_T_) obtained for singlet oxygen deactivation by AMSNPs-MOR was one order of magnitude lower than the morin rate constant reported in homogeneous solvents and lipid membranes. On the other hand, morin behaves as an antioxidant against hydroxyl radical generated *in situ*; nevertheless, NP presented also antioxidant properties by itself. Interestingly, adsorbed morin onto NP exhibits a synergic effect on the antioxidant property against hydroxyl radicals. This effect was increased when increasing the concentration of morin adsorbed, in the concentration range of work.

In summary, taken together the results suggest a complex adsorption process involving at least two ways; first, one monolayer irreversibly adsorbed like bonding-mode and then, presumably, multilayers in a piled up form physisorbed by electrostatic forces. The conjugation, can implicate an interaction between amino group from APTES with carboxyl and/or hydroxyl group on C ring of morin, leading to a resorcinol moiety accessible to react with different ROS or interact with 2´-OH group on the B ring.
